# A Systematic Study of Gene Mutations in Urothelial Carcinoma; Inactivating Mutations in *TSC2* and *PIK3R1*

**DOI:** 10.1371/journal.pone.0018583

**Published:** 2011-04-14

**Authors:** Gottfrid Sjödahl, Martin Lauss, Sigurdur Gudjonsson, Fredrik Liedberg, Christer Halldén, Gunilla Chebil, Wiking Månsson, Mattias Höglund, David Lindgren

**Affiliations:** 1 Department of Clinical Sciences, Oncology, Lund University, Skåne University Hospital, Lund, Sweden; 2 Department of Clinical Sciences, Urology, Skåne University Hospital, Malmö, Sweden; 3 Section of Urology, Växjö County Hospital, Växjö, Sweden; 4 Department of Laboratory Medicine, Clinical Chemistry, Lund University, Skåne University Hospital, Malmö, Sweden; 5 Department of Pathology, Helsingborg Hospital, Helsingborg, Sweden; 6 Center for Molecular Pathology, Department of Laboratory Medicine, Lund University, Skåne University Hospital, Malmö, Sweden; The University of Hong Kong, China

## Abstract

**Background:**

Urothelial carcinoma (UC) is characterized by frequent gene mutations of which activating mutations in *FGFR3* are the most frequent. Several downstream targets of FGFR3 are also mutated in UC, *e.g.*, *PIK3CA*, *AKT1*, and *RAS*. Most mutation studies of UCs have been focused on single or a few genes at the time or been performed on small sample series. This has limited the possibility to investigate co-occurrence of mutations.

**Methodology/Principal Findings:**

We performed mutation analyses of 16 genes, *FGFR3*, *PIK3CA*, *PIK3R1 PTEN*, *AKT1*, *KRAS*, *HRAS*, *NRAS*, *BRAF*, *ARAF*, *RAF1*, *TSC1*, *TSC2*, *APC*, *CTNNB1*, and *TP53*, in 145 cases of UC. We show that *FGFR3* and *PIK3CA* mutations are positively associated. In addition, we identified *PIK3R1* as a target for mutations. We demonstrate a negative association at borderline significance between *FGFR3* and *RAS* mutations, and show that these mutations are not strictly mutually exclusive. We show that mutations in *BRAF*, *ARAF*, *RAF1* rarely occurs in UC. Our data emphasize the possible importance of APC signaling as 6% of the investigated tumors either showed inactivating *APC* or activating *CTNNB1* mutations. *TSC1*, as well as *TSC2*, that constitute the mTOR regulatory tuberous sclerosis complex were found to be mutated at a combined frequency of 15%.

**Conclusions/Significance:**

Our data demonstrate a significant association between *FGFR3* and *PIK3CA* mutations in UC. Moreover, the identification of mutations in *PIK3R1* further emphasizes the importance of the PI3-kinase pathway in UC. The presence of *TSC2* mutations, in addition to *TSC1* mutations, underlines the involvement of mTOR signaling in UC.

## Introduction

Urothelial carcinoma (UC) of the bladder, the most common type of bladder cancer, is characterized by several gene mutations of which the most frequent is activating mutations in the *FGFR3* receptor protein. *FGFR3* mutations show a biased distribution of UC pathological subtypes in which low grade non-invasive tumors (Ta) shows the highest frequencies, close to 70%, whereas high grade and muscle invasive tumors (≥T2) show considerably lower frequencies, in the range of 10 to 15%. On the other hand, *TP53* mutations show an opposite pattern with high frequencies in muscle invasive tumors (∼50%) and low in non muscle invasive tumors (∼15%). This biased distribution of *FGFR3* and *TP53* mutations has led to the hypothesis that UC develops along two different pathways, a FGFR3 and a TP53 pathway, respectively [1], [2]. Alternative explanations for the frequent *TP53* mutations in muscle invasive tumors have however been put forward [3]. Several genes acting downstream of receptor tyrosine kinases (RTKs) have also been reported to be mutated in UC, *e.g. PIK3CA* [4], members of the *RAS* family [5], *BRAF* [6], and *AKT1* [7]. Another characteristic of UC is the frequent LOH on chromosome 9 and this chromosome is believed to harbor more than one tumor suppressor gene of importance for UC development. At least two such loci have been established, *CDKN2A* and *TSC1*. *CDKN2A* shows homozygous deletions in up to 30% of UC [8] whereas inactivating sequence mutations is seen to a lesser extent. *TSC1*, on the other hand, has been reported to be mutated in 16% of UC [4]. TSC1 is a negative regulator of the mTOR pathway, which is important for cell proliferation and frequently found activated in tumors [9] including UC [10], [11]. Notably, TSC1 is regulated by AKT1 and is therefore a potential downstream target of the FGFR3 signaling pathway. Additional proteins in this pathway include PIK3R1, PTEN and TSC2. PIK3R1 is a negative regulator of PIK3CA while PTEN is a negative regulator of AKT1. TSC2 forms a complex with TSC1 that functions as a negative regulator of the mTOR pathway. So far no mutation data on *PIK3R1* or *TSC2* in UC is available. Recent reports have also implicated the APC/CTNNB1 pathway in UC [12], [13], [14]. In the present investigation we aimed to further characterize the mutational landscape of UC. In a series of 145 tumors we performed mutation analyses of 16 genes, *FGFR3*, *PIK3CA*, *PIK3R1*, *PTEN*, *AKT1*, *KRAS*, *HRAS*, *NRAS*, *BRAF*, *ARAF*, *RAF1*, *TSC1*, *TSC2*, *APC*, *CTNNB1*, and *TP53*.

## Methods

### Tumors and isolation of nucleic acids

Urothelial tumors were collected by cold-cup biopsies from the exophytic part of the bladder tumor from 145 patients undergoing transurethral resection at the University Hospital of Lund, Sweden, between 2001 and 2005. For detailed patient information see Table S1. To increase the statistical power, the 145 series of samples were extended with 73 samples analyzed for *FGFR3*, *PIK3CA*, *TP53*, *HRAS*, *KRAS*, and *NRAS* mutations only (Table S1). Tumor pathology, including transurethral and cystectomy specimens, were reviewed by one pathologist (GC). Written informed consent was obtained from all patients and the study was approved by the Local Ethical Committee of Lund University. Genomic DNA was extracted using the DNeasy Tissue kit (Qiagen). Genomic DNA was amplified using the Illustra GenomiPhi V2 DNA Amplification Kit (GE Healthcare) before further processing.

### Mutation analysis

Coding regions in *FGFR3*, *PIK3CA*, *PIK3R1*, *PTEN*, *AKT1*, *KRAS*, *HRAS*, *NRAS*, *BRAF*, *ARAF*, *RAF1*, *TSC1*, *TSC2*, *APC*, *CTNNB1*, and *TP53* were selected and PCR-amplified using oligonucleotide primers (Table S2). All reactions were carried out in 96-well plates in a 40 ul mixture containing PCR buffer, 1.5 mM MgCl2, 0.2 mM DNTP, 0.5 µM each of the forward and reverse primers and 1 U TrueStart Taq polymerase (Fermentas, Helsingborg, Sweden) or Platinum Taq polymerase (Invitrogen, Carlsbad, CA). The reactions were heated to 94°C for 5 min, and subjected to 38–41 amplification cycles, followed by a final elongation step of 10 min at 72°C. Each cycle consisted of a denaturation step of 30 seconds at 94°C, an annealing step of 30 seconds, and an elongation step of 1 min at 72°C. The different annealing temperatures used in the different PCRs, as well as any deviations from the standard reaction mixture are listed in Table S2. All PCR-amplifications were carried out in a MBS Satellite Thermal Cycler (Thermo Scientific, Waltham, MA). Three ml of each PCR product were run on a precast 2% agarose gel (E-Gel 96 2% agarose GP, Invitrogen) and the remaining volume was purified on AcroPrep 96 filter plate Omega 10K (Pall, Ann Arbor, MI), according to the manufacturers protocol. The purified PCR products were then sequenced using the BigDye terminator v1.1, or 3.1 cycle sequencing kit (Applied Biosystems, Foster City, CA) on a 3130×l Genetic Analyzer (Applied Biosystems). Sequence traces were analyzed using the SeqScape v2.5 software (Applied Biosystems), and all sequence variations were validated by re-sequencing independent PCR products. A change in the DNA sequence was considered to be a mutation when it changed the amino acid sequence of the encoded protein or affected a known splice acceptor or donor site. Sequence changes reported as single nucleotide polymorphisms (SNPs) according to Database of Single Nucleotide Polymorphisms (dbSNP) at NCBI, were omitted from the analysis. All gene mutations not previously described were validated by sequencing of blood samples obtained from the same patient when available (Table S3). Previously not described mutations were analyzed using the PolyPhen 2 predictor tool [15].

### Statistical analysis

Chi-2 analyses were used to establish significant differences in proportions between groups. A hypergeometric test was used to determine if the observed numbers of double mutations was significantly different from what is expected from a resample distribution assuming independence.

## Results

**Figure 1:**
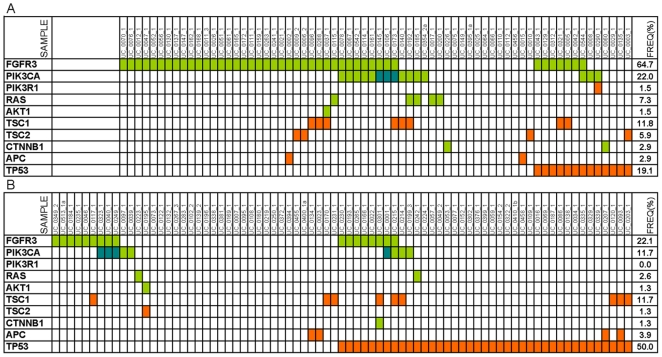
Distribution of identified mutations. In **A**) low grade (G1/G2) tumors and in **B**), high grade (G3) tumors. Red squares indicate inactivating mutation. Green squares indicate activating mutation. For *PIK3CA*, dark green squares indicate kinase domain mutations and light green helical domain mutations. At the right, mutation frequencies are given for each gene in the respective tumor grades.

**Figure 2:**
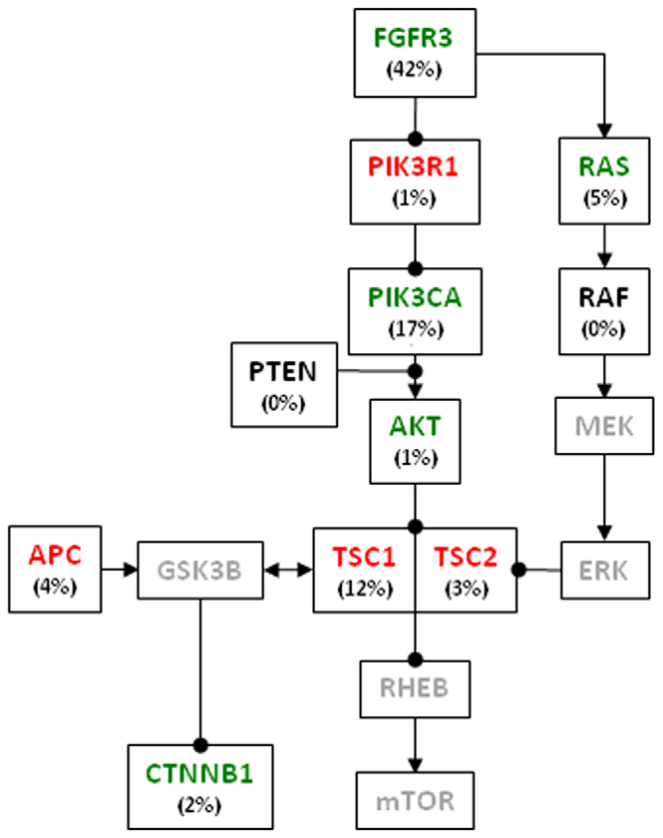
Schematic representation of relationships and mutation frequencies among the investigated genes. Arrowheads, positive regulation; filled circles, negative regulation; gene names in green, genes showing activating mutations; gene names in red, genes showing inactivating mutations; gene names in black, genes with no detected mutations in the present investigation; gene names in gray, genes not investigated. *TP53* is not included in the graph. The mutation frequencies (%) given are based on the 145 samples investigated for each gene.

We screened 145 UCs for sequence mutations in a total of 16 genes, *FGFR3*, *PIK3CA*, *PIK3R1*, *PTEN*, *AKT1*, *KRAS*, *HRAS*, *NRAS*, *BRAF*, *ARAF*, *RAF1*, *TSC1*, *TSC2*, *APC*, *CTNNB1*, and *TP53*. In Figure 1 the results are summarized and the cases are grouped according to tumor grade. The same data organized according to tumor stage is provided as Figure S1. Mutation frequencies are also summarized in Figure 2, along with possible interactions between the investigated genes. Detailed information on all identified mutations is given in Table S3.

For FGFR3 we sequenced exons 7, 10, and 15. Mutations in these exons correspond to 96% of the *FGFR3* mutations seen in tumors of the urinary tract according to the COSMIC database (www.sanger.ac.uk/genetics/CGP/cosmic). We detected mutations at 7 different amino acid positions, all previously described. Mutations were seen in 65% of low grade (G1/G2) and in 22% of high grade tumors (Table 1), and in 63%, 39%, and 22% of Ta, T1, and ≥T2, respectively (Figure S1). As expected, the *FGFR3* mutation frequency was significantly higher in low grade tumors (p<0.0001, Chi2 test,) and in non-muscle invasive (NMI) compared to muscle invasive (MI) cases, 55% and 22% respectively, (p<0.0002). These results are in line with previous investigations [16], [17], [18] and clearly show that *FGFR3* mutations are associated with low grade low stage tumors and with NMI tumors in particular.

**Table 1:** Mutation frequencies.

	Ta (%) (n = 54)	T1 (%) (n = 36)	≥T2 (%) (n = 54)	G1/G2 (%) (n = 68)	G3 (%) (n = 77)	Total (n = 145)	Total (%)	Extended (n = 218)
FGFR3	63.0	38.8	22.2	63.2	22.1	61	42.1	82 (37.6%)
PIK3CA	22.2	16.6	9.3	22.1	11.7	24	16.6	37 (17.0%)
PIK3R1	0	2.8	0	1.5	0	1	0.7	
RAS	3.7	11.1	1.9	7.4	2.6	7	4.8	10 (4.6%)
AKT1	0	2.8	1.9	1.5	1.3	2	1.4	
TSC1	11.1	16.7	9.3	11.8	11.7	17	11.7	
TSC2	1.9	8.3	1.9	5.9	1.3	5	3.4	
APC	1.9	0	9.3	2.9	5.2	6	4.1	
CTNNB1	1.9	5.6	0	2.9	1.3	3	2.1	
TP53	14.8	47.2	51.9	19.1	50.6	52	35.9	73 (33.5%)

We next performed mutation analysis of *TP53* and a total of 52 *TP53* mutations (36%) were detected. As expected, we found a significant difference in frequency between high grade (51%) and low grade tumors (19%) (p<0.0001). We then tested for possible negative or positive associations between *TP53* and *FGFR3* mutations. To increase the power we included mutation data for an additional 73 cases increasing the number of cases from 145 to 218 and a negative association was observed (p = 0.0085, hypergeometric test). This association is however lost when G1/G2 and G3 cases are tested separately, p = 0.39 and p = 0.43, respectively.

*PIK3CA* was screened for mutations in exons 9 and 20 that contain the hotspot positions in which close to all activating mutations occur [19]. A total of 37 (17%) mutated cases were detected in the extended series of tumors (n = 218). A significantly higher proportion of PIK3CA mutations was seen in Ta cases compared to T1 (p<0.05, Chi-2 test), but not between T1 and ≥T2, or between NMI and MI cases. *PIK3CA* mutations was also associated with low grade (p<0.01). The data also indicated a possible association between *FGFR3* and *PIK3CA* mutations, with 23 detected double mutations and 14 expected. To further investigate this we added data for 92 UCs previously published by Platt et al. [4], 87 published by Lopez-Knowles et al. [20], and 257 by Kompier et al. [18]. In this combined dataset (n = 654) a significant association between *FGFR3* and *PIK3CA* mutations could be established (hypergeometric test, p<2×10^−7^, 95 observed double mutants and 68 expected). We then tested for possible negative or positive associations between *PIK3CA* and *TP53* mutations but no significant association was observed (p = 0.066, hypergeometric test, n = 218). We also performed mutation analyses of two modulators of PIK3CA activity, PTEN and PIK3R1. All exons of *PTEN* were sequenced in the 145 tumors but no mutations were identified. For *PIK3R1* we sequenced exons 12, 14, and 15 and found one case with mutation.

We next sequenced the exons covering codons 12, 13, and 61 in *HRAS*, *KRAS*, and *NRAS* and detected a total of six mutations in *HRAS*, four in *KRAS*, and none in *NRAS* (n = 218). The overall frequency of *RAS* mutations was 5%, which is somewhat lower than what has been reported in recent investigations using DNA sequencing [4], [18], [21]. We further tested for a possible negative association between *RAS* and *FGFR3* mutations but only found borderline statistical support for such an association (p = 0.059, hypergeometric test, n = 218).

BRAF has been shown to be activated by point mutations in several different tumor types [22] with the most frequent mutations located in exons 11 and 15. *BRAF* belongs to a gene family that also includes *ARAF* and *RAF1*, which mediates signals from RAS to downstream targets. As *RAF* mutations, in analogy with *RAS* mutations, may show tumor type specificity, we sequenced exons 11 and 15 in *BRAF* and the equivalent exons in *ARAF* (exons 10 and 13) and *RAF1* (exons 11 and 14). In addition, *RAF1* exon 7 was sequenced since activating mutations in this exon has been described in the Noonan Syndrome [23]. The equivalent exon was also sequenced in *ARAF*. No mutations were however detected in any of the *RAF* genes.

AKT1 is a major downstream target of PIK3CA and is known to play a key role in the regulation of cell cycle progression, survival, and mTOR signaling. *AKT1* was recently shown to have activating mutations in exon 4 in UC [7]. We sequenced the complete exon 4 of *AKT1* in our series of 145 tumors and found 2 tumors with an E17K mutation (1%) (Figure 1), associated with increased and constitutive kinase activity of AKT1 under conditions of growth factor withdrawal [7].

We screened all 21 coding exons of *TSC1* and detected a total of 17 mutations (13%) including truncating, missense, and splice site mutations. In two additional tumors a substitution of asparagine to serine was seen at amino acid position 762. Both changes were however also detected in blood DNA from the respective patients and hence were considered to be naturally occurring polymorphisms. There was no difference in frequency between low and high grade tumors and *TSC1* mutations were seen in *FGFR3*, *PIK3CA*, and in *TP53* mutated cases. TSC1 functions together with TSC2 as an inhibitor of mTOR by maintaining the mTOR activator RHEB in an inactive state. We therefore sequenced all coding exons of *TSC2* and found a total of 5 mutations in 145 samples (3%). The possible impacts of these mutations on protein function were investigated by the PolyPhen software. The software predicted a damaging effect for all four missense mutations, the fifth being a 1 bp frame shift deletion. TSC2 mutations were seen in both high and low grade tumors and none of the *TSC2* mutated cases showed concomitant *TSC1* mutations. We also tested for possible associations between *TSC1* mutations, or *TSC1* and *TSC2* combined, and mutations in *FGFR3* or *TP53*. No such association was however found (p>0.25 in all comparisons, hypergeometric test, n = 145).

We sequenced exon 16 in *APC*, known to harbor the majority of *APC* mutations [24], and exon 3 of *CTNNB1* covering the phosphorylation sites that control ubiquitination and degradation of CTNNB1. We detected 6 APC mutations in 145 cases (4%), with no difference between low and high grade tumors. *CTNNB1* mutations were seen in 3 cases (2%). We found *APC/CTNNB1* mutations in both *FGFR3* and *TP53* wild type and mutated cases, indicating that activation of the APC/CTNNB1 signaling pathway occur independent of *FGFR3* and *TP53* mutations. All detected *APC* mutations were missense mutations. This is in line with Kastritis et al. [12], who also noted an underrepresentation of truncating mutations compared to the mutation spectrum in colorectal cancer. In fact the difference is highly significant. Our data combined with the data of Kastritis et al. show a significantly lower frequency of truncating mutations when compared with data obtained for colorectal cancer from the COSMIC database (p<0.0001, Chi-2).

## Discussion

The aim of the present investigation was to further establish mutation frequencies for genes commonly mutated in UC, and to investigate possible positive or negative associations between mutations. To accomplish this we sequenced 14 genes, previously shown to be mutated in UC, in 145 cases. *FGFR3*, *PIK3CA*, *HRAS*, *KRAS*, *NRAS*, and *TP53* were sequenced in an additional 73 cases. In addition, we sequenced *PIK3R1* and *TSC2* as no mutation data has been published for these two genes in UC. The observed high frequencies of *FGFR3*, *TP53*, and *PIK3CA* mutations were as expected. We detected a positive association between *FGFR3* and *PIK3CA* activating mutations as reported [18], [20], and by combing our data with previously published data we could confirm that this association is highly significant. Two of our cases showed double mutations in *PIK3CA*; one case with a HD and a KD domain mutation, and one case with two mutations in the HD domain (E542K E545K). If these double mutations had occurred in separate alleles could not be determined as the electropherograms indicated heterozygous mutations. Irrespectively, the repeated observations of *PIK3CA* double mutated tumors and cell lines [4], [18], [25], [26] indicate an additive effect of multiple *PIK3CA* mutations.

PTEN acts as negative modulator of PIK3CA and is mutated in many tumor types as well as in UC [27], [28]. No *PTEN* mutations were however detected in the present set of tumors, a finding in line with previous reports of a low *PTEN* mutation frequency in UC (2%, n = 88) [29], [30]. This does however not exclude PTEN as an important factor in bladder cancer development since *PTEN* frequently shows reduced expression [31] as well as homozygous deletion in UC [29]. As mentioned, PIK3R1 also acts as a negative modulator of PIK3CA and has been shown to have tumor suppressor activity [32]. We found one case with a mutation after sequencing exons 12, 14, and 15, the most frequently mutated regions in this gene [33]. These exons cover the major part of the PIK3R1 iSH2 domain that interacts with PIK3CA. The frequent down regulation of PTEN expression and the here reported mutational inactivation of PIK3R1 further emphasized the importance of PIK3CA activity in UC.

Published data on *RAS* mutation is highly variable with respect to frequency, the distribution of codon 12 and 13 vs. codon 61 mutations in *HRAS*, and the proportion between *HRAS*, *KRAS*, and *NRAS* mutations [5], [34], [35], [36]. The divergent results may partly be attributed to differences in methods used and partly to differences in sample selection. In the present investigation *HRAS* mutations dominated over *KRAS* mutations and *HRAS* codon 61 mutations were much more frequent than codon 12 mutations, which is in line with most previous investigations.

It has been suggested that *RAS* mutations may substitute for *FGFR3* mutations and that *RAS* and *FGFR3* mutations therefore are mutually exclusive [21]. This hypothesis is however weakened by the fact that samples with concomitant *FGFR3* and *RAS* mutations were identified in the present investigation as well as in the studies by Platt et al. and Kompier et al. [4], [18]. These findings may either be explained by intra-tumor heterogeneity as suggested by Platt et al., or that the biological consequences of activating *FGFR3* and *RAS* mutations do not overlap completely and therefore double mutants may result in an additive but small selective advantage. Irrespective if these mutations are mutually exclusive or not, the evidence for a strong negative association between these two genes is compelling [4], [18], [21]. BRAF operates downstream of the RAS proteins and has been shown to be mutated in several tumor types [22]. We did not detect any mutations in any of the RAF gene family members. Hence, our data show that the previously reported low frequency or absence of *BRAF* mutations is not substituted for by frequent mutations in either *ARAF* or *RAF1*. Consequently, *RAF* mutations are not as central for UC development as *FGFR3*, *PIK3CA*, and *RAS* mutations.

Few and highly divergent mutation frequencies of *APC* and *CTNNB1* have been reported for UC [12], [13], [37], [38]. Stoehr et al. found no *APC* or *CTNNB1* mutations in 99 investigated cases, which is in stark contrast to Kastritis et al. who found 11 *APC* mutations in 70 cases (16%), but no *CTNNB1* mutations in 35 investigated cases. Shiina et al., on the other hand, identified a total of 4 *CTNNB1* mutations in 64 UC cases (6%). Our results showing APC mutations in 4% and *CTNNB1* mutations in 2% of the cases are fully compatible with the combined published data resulting in overall mutation frequencies of 6.5% for *APC* and 2.0% for *CTNNB1*. As *APC* and *CTNNB1* mutations were not seen together the frequency of APC/CTNNB1 pathway alteration was 6% in our data. As Kastritis et al. [12], we found a strong bias towards missense mutations, in contrast to truncating mutations, in *APC* and we could show that the mutation spectrum in UC differs significantly from the spectrum seen in colorectal cancer.

We detected a total of 17 cases (12%) with TSC1 mutations in our data, which is close to the previously reported frequency of 16% [4]. *TSC1* is an established tumor suppressor gene in UC [4] that exert most of its regulatory function in a complex with TSC2. Consequently, we also screened *TSC2* for mutations and detected 5 mutations in 145 cases (3%). This is a slightly higher frequency than what has been reported for other investigated solid tumors; CNS tumors 0.6%, lung cancers 0.9%, ovarian carcinomas 0.6% (COSMIC database). TSC2 acts as a dimer together with TSC1 by regulating mTOR through RHEB. The activity of TSC1/2 is, in turn, regulated by input from several upstream regulators [39] making the TSC1/2 a hub for upstream signals funneled to mTOR. The combined frequency of *TSC1/TSC2* mutations of 15% indicates that a substantial proportion of UC tumors may show activation of mTOR through *TSC1* or *TSC2* mutational inactivation.

All in all, the present investigation emphasizes FGFR3, PIK3CA/AKT1, and TSC1/TSC2 as important nodes in intracellular signaling of transformed urothelial cells. To what extent FGFR3 activation may be directly linked to mTOR activation remain to be elucidated; we note that *FGFR3* and *PIK3CA* mutations show a strongly skewed distribution between low and high grade tumors whereas *TSC1/TSC2* mutations were seen in both categories at almost equal frequencies. Importantly, the detected alterations in the APC/CTNNB1 signaling pathway may also influence mTOR activity since GSK3B, an important member of the APC/CTNNB1 signaling pathway, is one of many modulators of TSC1 [40], [41]. There is further evidence for a crosstalk between APC/CTNNB1 and TSC1/TSC2 since wild type, but not inactive or mutated TSC2, regulates CTNNB1 negatively at the level of the CTNNB1 degradation complex [42]. In conclusion our data underscore the possible importance of mTOR activity in the development of UC. As mTOR activity is tractable for drug treatment [43], [44], and may be a possible target for various treatment regimes [43], [45], [46], [47], future investigations should be directed specifically towards mTOR activity in UC.

## Supporting Information

Figure S1 **Distribution of mutations in non-invasive Ta tumors (A), in T1 tumors (B) and in (C) muscle-invasive tumors (≥T2).** Red squares indicate inactivating mutation. Green squares indicate activating mutation. For *PIK3CA*, dark green squares indicate kinase domain mutations and light green helical domain mutations. At the right mutation frequencies are given for each gene in the respective tumor stages.
(TIF)

Table S1 Clinical features and mutation data.
(XLS)

Table S2 Primers and PCR conditions.
(XLS)

Table S3 Mutation details.
(XLS)
