# Supplementary figures and images for: A Systematic Study of Gene Mutations in Urothelial Carcinoma; Inactivating Mutations in TSC2 and PIK3R1
Source: PLoS One. 2011 Apr 14;6(4):e18583. doi: 10.1371/journal.pone.0018583 (PMC3077383; doi:10.1371/journal.pone.0018583)

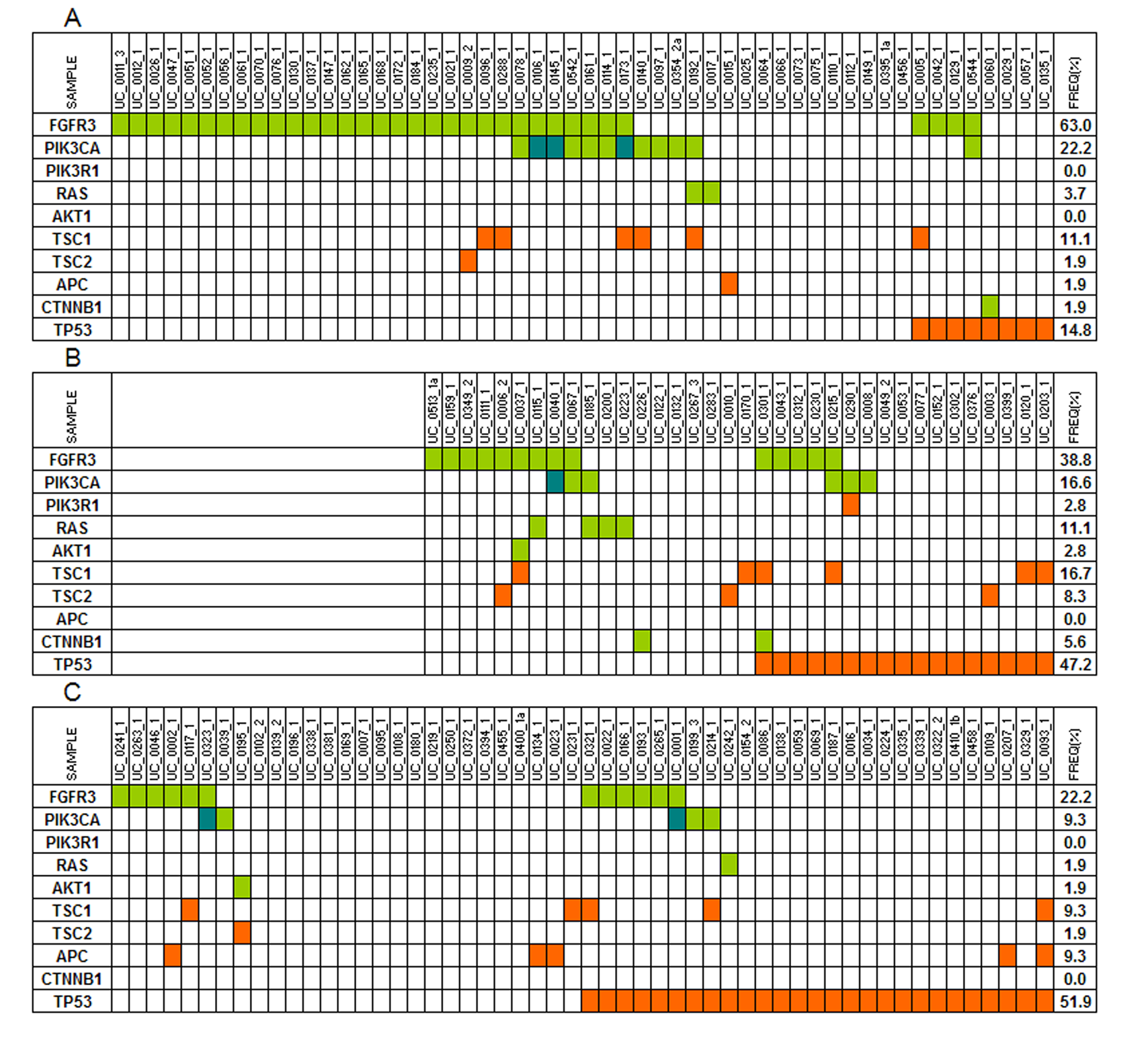

Supplement: Figure S1 — Distribution of mutations in non-invasive Ta tumors (A), in T1 tumors (B) and in (C) muscle-invasive tumors (≥T2). Red squares indicate inactivating mutation. Green squares indicate activating mutation. For PIK3CA, dark green squares indicate kinase domain mutations and light green helical domain mutations. At the right mutation frequencies are given for each gene in the respective tumor stages. (TIF) [file pone.0018583.s001.tif]
